# The immune inflammation factors associated with disease severity and poor prognosis in patients with COVID-19: A retrospective cohort study

**DOI:** 10.1016/j.heliyon.2023.e23583

**Published:** 2023-12-12

**Authors:** Yanli Kang, Shifa Lu, Ruifang Zhong, Jianbin You, Jiahao Chen, Ling Li, Rongbin Huang, Yanyan Xie, Falin Chen, Jinhua Chen, Liangyuan Chen

**Affiliations:** a Department of Clinical Laboratory, Fujian Provincial Hospital, Shengli Clinical Medical College of Fujian Medical University, Fuzhou, China; b Department of Clinical Laboratory, JianOu Municipal Hospital of Fujian Province, Nanping, China; c Department of Clinical Laboratory, The Second Affiliated Hospital of Fujian University of Traditional Chinese Medicine, Fuzhou, China

**Keywords:** COVID-19, Immunological characteristics, IL-6, IL-10, CD8^+^

## Abstract

Coronavirus disease 2019 (COVID-19) is associated with immune dysregulation and cytokine storm. It is essential to explore the immune response characteristics of peripheral circulation in COVID-19 patients to reveal pathogenesis and predict disease progression. In this study, the levels of total immunoglobulins (IgG, IgM, IgA), complement (C3, C4)，lymphocyte subsets (CD3^+^ cell，CD4+ cell，CD8+ cell, NK cell, CD19^+^ cell and CD45^+^ cell) and cytokines (IL-2, IL-4, IL-5, IL-6, IL-8, IL-10, IL-17, IL-12p, IL-1β, TNF-α, IFN-α and IFN-γ) were retrospectively analyzed in COVID-19 patients. A total of 513 patients were enrolled in this study, cases were distributed according to clinical status as mild or moderate (n = 212), severe survivors (n = 197) and severe non-survivors (n = 104). IL-6, IL-8, IL-10 and IFN-γ were increased in severe patients compared with non-severe patients, despite decreased CD45^+^ cell, CD3^+^ cell, CD4^+^ cell, CD8^+^ cell, CD19^+^ cell, and NK cell. Compared with severe survivors, the levels of L-6, IL-8 and IL-10 in non-survivors increased significantly, and levels of C3, CD45^+^ cell, CD3^+^ cell，CD4+ cell，CD8+ cell, and NK cell decreased. Moreover, age, IL-8, IL-10, CD8+cells and NK cell were independent risk factors for the severity of COVID-19. Multivariable regression showed increasing odds ratio of in-hospital death associated with tumor, older age, higher IL-8 level, and decreasing odds ratio of in-hospital death associated with increased levels of CD8+cell and NK cell. Finally, patients with tumor, or high IL-6 or high IL-10 expression and lower CD8^+^ or lower NK levels exhibited a significantly shorter survival time. In conclusion, our study provides findings of the immunological characteristics associated with disease severity to predict the progression of COVID-19. The immune inflammation factors, such as IL-6, IL-8, IL-10, CD8^+^ cell and NK cell, could serve as excellent biomarkers for monitoring or predicting COVID-19 progression therapeutic to COVID-19 patients.

## 1 Introduction

Coronavirus disease 2019 (COVID-19), caused by severe acute respiratory syndrome coronavirus 2 (SARS-CoV-2), continues to ravage the globe. The world health organization (WHO) reported that, as of February 12, 2023, there have been an estimated 755,385,709 confirmed positive cases and 6,833,388 deaths worldwide, and the number of cases is still climbing [1]. Although the symptoms and signs in most patients with COVID-19 patients are usually mild to moderate, about 15–20 % of cases progress to severe interstitial pneumonia, and even 5 % of individuals evolve into serious multiple complications, such as acute respiratory distress syndrome (ARDS), septic shock, and multiple organ failure [2,3]. Existing evidence reveals that the immune response dysregulation in host plays a potential role in the onset, disease evolution, and severity of COVID-19 [[4], [5], [6]].

The innate and acquired immune responses of the body are activated after infection with SARS-CoV-2, but excessive inflammatory responses can also lead to surrounding or even systemic tissue damage [7,8]. Lymphopenia is a typical laboratory abnormality observed during SARS-CoV-2 infections, particularly in severe and critical patients [9]. In contrast, neutrophils were significantly increased and aggregated at the site of infection, and also induced cytokine storm by activating additional inflammatory signals through the release of pro-inflammatory mediators [10]. As one of the principal protagonists in innate and acquired immunity, complement plays a controversial role in SARS-CoV-2 infection. While it may be effective in helping to control this infection in many asymptomatic individuals and even patients with mild symptoms, and it also may promote neutrophil activation and cause cytokine storms due to its potent pro-inflammatory effect [11].

Unfortunately, there are still missing puzzle pieces regarding the aberrant immune response in the progression of COVID-19 remain largely unknown. Therefore, it is critical to explore the immune response characteristics of peripheral circulation in COVID-19 patients to better understand the pathogenesis as well as predict disease progression. In the present study, we evaluated the expression of inflammatory cytokines, lymphocyte count, cytokines and complement in different populations to provide important cues for comprehending the underlying molecular mechanisms of the immune response dysregulation by SARS-CoV-2 infection. The study would provide potential biomarkers for predicting COVID-19 progression.

## 2 Materials and methods

### 2.1 Study population

This is a single center retrospective case analysis series. From December 21, 2022 to January 21, 2023, a total of 570 inpatient patients diagnosed with COVID-19 (by PCR or antigen rapid test kit) have been hospitalized at Fujian Provincial Hospital. Based on the following inclusion criteria and exclusion criteria, excluding 4 cases under the age of 18, 50 cases missing key information and 3 cases transferred to other hospitals with no end point, 513 cases were enrolled for statistical analysis ultimately. Inclusion criteria: (1) adults ≥ 18 years old, (2) COVID-19 infection confirmed through polymerase chain reaction testing or COVID-19 antigen testing, (3)receiving inpatient care, (4) the outcome was discharge or death. Exclusion criteria: (1) children and young adults (17 years and under), (2) people with missing data of length of hospital stay because they still in hospital till January 21, 2023, (3) people transferred to other hospitals with no end point.

### 2.2 Definitions

All the hospitalized patients were subdivided into mild or moderate or severe or critical according to the Guidance for Corona Virus Disease 2019 (10th edition) released by the National Health Commission of China.

The classification criteria were listed below.

(1) Mild type: the main manifestation of above respiratory tract infection, such as dry throat, sore throat, cough, fever, etc.
(2) Moderate type: persistent high fever >3 days or (and) cough, shortness of breath, etc., but respiratory rate (RR) < 30 times/min, oxygen saturation >93 % when breathing at rest. Imaging shows the characteristic manifestations of COVID-19 pneumonia.
(3) Severe type: respiratory rate ≥ 30 breaths/min, resting blood oxygen saturation ≤ 93 %, partial pressure of arterial blood oxygen/fraction of inspired oxygen ≤ 300 mm Hg, clinical symptoms gradually worsening, and pulmonary imaging showing that lesions had progressed more than 50 % within 24–48 h.
(4) Critical type: respiratory failure, defined as the need for mechanical ventilation, shock, other organ failure requiring ICU monitoring or treatment.

In our study, 212 patients with mild or moderate COVID-19 were categorized as the non-severe group and 301 patients with severe or critical COVID-19 were categorized as the severe group. Ulteriorly, according to the in-hospital outcomes, the severe cases were categorized as survivors (n = 197) and non-survivors (n = 104). This study was reviewed and approved by the Ethical Committee of Fujian Provincial Hospital with the approval number: KY2021-03-013.

### 2.3 Data collection

We focused on clinical history at presentation, immunological investigations on the day of admission before treatment, length of hospital stay and in-hospital outcomes. The date of admission to hospital for each patient enrolled in the study, as well as the age, gender, and comorbidities (e.g. hypertension, diabetes mellitus (DM), chronic cardiovascular disease, chronic pulmonary disease, chronic kidney disease, and tumor) were documented. In addition, the data of immunological investigations were divided to three groups: inflammatory cytokines (IL-2, IL-4, IL-5, IL-6, IL-8, IL-10, IL-17, IL-12p, IL-1β, TNF-α, IFN-α, IFN-**γ**), lymphocyte subset (CD3^+^ cell, CD4^+^ cell，CD8+ cell, NK cell, CD19^+^ cell and CD45^+^ cell), and immunoglobulin (IgG, IgM, IgA) and complement factors (C3, C4). Inflammatory cytokines levels were measured by Cytokine combined detection kit (Ceger, JiangXi, China) in BeamCyte flow cytometer (BeamCyte, Changzhou, China) according to the manufacturer's instructions. The kit is based on immunofluorescence technology. In brief, the capture microspheres in the kit are coated with L-1β, IL-2, IL-4, IL-5, IL-6, IL-8, IL-10, IL-12p70, IL-17, IFN-α, IFN-γ and TNF-α specific antibodies, respectively, which can bind to these cytokines specifically. These conjugations are then combined with PE-labeled fluorescence detection reagents. Finally, the levels of cytokines were established by detecting fluorescence intensity. Lymphocyte subset levels were measured by BD Multitest 6-colour TBNK reagent and BD Trucount tubes (Becton Dickinson, CA, USA) according to the manufacturer's instructions. A lyse-no-wash protocol was used. Briefly, whole blood was stained with the Multitest 6-colour TBNK reagent (containing a cocktail of the following antibodies: CD3 FITC, CD16 and CD56 PE, CD45 PerCp Cy5.5, CD4 PE-Cy7, CD19 APC, and CD8 APC-Cy7). After the first incubation (15 min at room temperature in the dark), BD FACS lysing solution (Becton Dickinson, CA, USA) was added to the tubes. After a second incubation (15 min at room temperature in the dark), samples were measured on a BD FACSCanto II and analyzed with the BD FACSCanto clinical software (Becton Dickinson,CA, USA). The levels of IgG, IgA, IgM, C3 and C4 were calculated by the IgG/IgA/IgM/C3/C4 Flex Reagent Cartridge kit (Siemens Healthineers, Germany) and a BNII specific protein analyzer (Siemens Healthineers, Germany). In brief, the levels of immunoglobulin and complement factors were measured in nephelometric immunoassay, based on the measurement of an antigen-antibody reaction.

### 2.4 Statistical analysis

Statistical analyses were performed with SPSS 22 and GraphPad Prism 5.0. The data conforming to normal distribution are expressed by mean ± SEM, comparison between two groups with unpaired *t*-test. The data conforming to non normal distribution are described as medians and 25th–75th percentile quartile intervals (IQRs) and are compared using the Mann-Whitney U or Kruskal Wallis test. Categorical variables are expressed as numbers and percentages [n (%)], and are compared using contingency table analysis and χ2 tests or Fisher's exact test. Receiver operating characteristic (ROC) curve, and the areas under the curve (AUCs) were performed to evaluate the diagnostic value of significant parameters. The binary logistic regression analysis were performed to evaluate the effect of the immunological factors on COVID-19 severity or in-hospital outcomes in severe. Overall 30-day survival, in which the start date was considered the date of admission, and the end date was the date of death (for failures), was compared by the Kaplan-Meier method and log-rank test. A p-value of less than 0.05 was considered statistically significant.

## 3 Results

### 3.1 Clinical characteristics of COVID-19 patients and in-hospital outcomes in the severe group

The clinical characteristics of 513 patients with COVID-19 are listed in Table 1. Overall, approximately 58.7 % of inpatient patients with COVID-19 develop a severe case, and of those severe cases 34.6 % die. Severe patients were older than non-severe patients (74 [IQR60-82]) vs. 78 [IQR69-84]), and non-survivors were older than survivors. Age difference between the cases was statistically significant. The median length of hospital stay in non-severe group was 9 days (IQR 6–13), while 13 days (IQR 9–23) in severe group, and those of survivors and non-survivors were 14 days (IQR 10–23) and 12 days (IQR 6–23), respectively. The proportion of participants with non of observed comorbidities was significantly lower in severe than non-severe, but significant differences between survivors and non-survivors did not exist.

**Table 1:** Clinical characteristics of COVID-19 patients and in-hospital outcomes in the severe group.

Variables	Disease severity		*P*	In-hospital outcomes in severe		*P*
	Non-severe	Severe		Survivors	Non-survivors	
Overall	212 (41.3 %)	301 (58.7 %)		197 (65.4 %)	104 (34.6 %)	
Gender			0.0612			0.0963
Male	126 (59.4 %)	204 (67.8 %)		125 (63.5 %)	76 (73.1 %)	
Female	86 (40.6 %)	97 (32.2 %)		72 (36.5 %)	28 (26.9 %)	
Age (yr)	74 (60–82)	78 (69–84)	**0.0009**	75 (68–84)	80 (72–86)	**0.0071**
Length of hospital stay	9 (6–13)	13 (9–23)	**< 0.0001**	14 (10–23)	12 (6–23)	**0.0181**
Non of observed comorbidities	45 (21.2 %)	33 (11.0 %)	**0.0017**	23 (13.8 %)	10 (9.6 %)	0.6994
Comorbidities						
Hypertension	113 (53.3 %)	190 (63.1 %)	**0.0288**	128 (65.0 %)	62 (59.6 %)	0.3808
Diabetes	79 (37.3 %)	124 (41.2 %)	0.4095	79 (61.7 %)	45 (43.3 %)	0.6235
Chronic cardiovascular disease	54 (25.5 %)	106 (33.6 %)	**0.0203**	62 (31.5 %)	44 (42.3 %)	0.0755
Chronic pulmonary disease	14 (6.6 %)	37 (12.3 %)	**0.0363**	26 (13.2 %)	11 (10.6 %)	0.5827
Chronic kidney disease	40 (18.9 %)	69 (22.9 %)	0.2757	42 (21.3 %)	27 (26.0 %)	0.3885
Tumor	35 (16.5 %)	54 (17.9 %)	0.7232	27 (13.7 %)	27 (26.0 %)	**0.0112**

Values are median (IQR) or n (%).

The comorbidities of hypertension, chronic cardiovascular disease and chronic pulmonary disease had a higher incidence in severe cases, and the difference was significant. It's worth noting the incidence of tumor was a statistically significant difference between non-survivors and survivors, rather than non-severe cases and severe cases.

### 3.2 The immunological investigations on admission in patients with COVID-19

The data of immunological investigations were divided to three groups: inflammatory cytokines, lymphocyte subset, and immunoglobulin and complement factors. The characteristics of the three groups were shown in Table 2, Table 3, Table 4, respectively. In the inflammatory cytokines group (Table 2), the levels of IL-6, IL-8, IL-10 and IFN-γ in severe patients were higher than non-severe patients. Serum levels of IL-6, IL-8 and IL-10 were higher in non-survivors than in survivors, and the similar differences were not found for IFN-γ. In the lymphocyte subset group (Table 3), the amount of lymphocyte subset (CD45^+^ cell, CD3^+^ cell，CD4+ cell，CD8+ cell, and NK cell) not only were significantly lower in severe patients than non-severe patients, but also lower in non-survivors than survivors. But the level of CD19^+^ cell was a statistically significant difference between non-severe cases and severe cases, rather than non-survivors and survivors. In the immunoglobulin and complement factors group (Table 4), there was not any difference between the severe patients and non-severe patients, and only C3 was significantly different between non-survivors and survivors.

**Table 2:** The inflammatory cytokines levels evaluated among COVID-19 patients.

Variables	Range of normal value	Disease severity		*P*	In-hospital outcomes in severe		*P*
		Non-severe	Severe		Survivors	Non-survivors	
Overall							
IL-2 (pg/mL)	0–5.71	0.87 (0.43–2.04)	0.82 (0.25–1.75)	0.6896	0.74 (0.33–1.75)	10.02 (0.14–22.14)	0.8324
<5.71		41/43 (95.4 %)	80/83 (96.4 %)	1.0000	45/47 (95.8 %)	35/36 (97.2 %)	1.0000
IL-4 (pg/mL)	0.00–3.00	3.82 (2.01–6.91)	4.86 (2.10–7.87)	0.4237	4.86 (2.03–7.69)	4.92 (2.20–8.50)	0.7190
<3.0		14/42 (33.3 %)	25/82 (30.5 %)	0.8386	14/46 (30.4 %)	11/36 (30.6 %)	1.0000
IL-5 (pg/mL)	0.00–3.10	0.76 (0.40–1.08)	0.83 (0.42–1.09)	0.7018	0.83 (0.44–1.04)	0.82 (0.34–1.12)	0.9144
<3.1		42/42 (100 %)	80/82 (97.6 %)	1.0000	45/46 (97.8 %)	35/36 (97.2 %)	1.0000
IL-6 (pg/mL)	0.00–5.30	5.40 (2.72–14.74)	8.86 (3.74–45.60)	**0.0082**	6.38 (3.05–14.74)	38.35 (8.09–542.90)	**<0.0001**
<5.30		31/63 (86.1 %)	39/109 (35.8 %)	0.1072	30/65 (41.2 %)	9/44 (20.5 %)	**0.0079**
IL-8 (pg/mL)	0.00–20.60	27.38 (20.75–39.63)	35.52 (28.97–52.63)	**0.0003**	32.60 (25.89–41.73)	43.07 (31.95–139.30)	**0.0007**
<20.60		10/42 (23.8 %)	4/82 (4.9 %)	**0.0048**	3/46 (6.5 %)	1/36 (2.8 %)	0.6272
IL-10 (pg/mL)	0.00–4.91	3.28 (1.77–5.70)	5.40 (3.26–11.58)	**0.0004**	4.86 (3.03–8.50)	6.59 (3.85–21.65)	**0.0339**
<4.91		30/42 (71.4 %)	35/82 (42.7 %)	**0.0023**	24/46 (52.2 %)	11/36 (30.6 %)	0.0717
IL-17 (pg/mL)	0.00–20.60	8.29 (0.45–18.23)	10.02 (0.14–22.14)	0.6069	9.18 (0.27–23.51)	12.80 (0.11–19.72)	0.9887
<20.60		35/42 (83.3 %)	60/82 (73.2 %)	0.2645	32/46 (69.6 %)	28/36 (77.8 %)	0.4592
IL-12p70 (pg/mL)	0.00–3.40	1.90 (0.10–4.05)	2.51 (0.10–5.32)	0.3593	2.32 (0.10–5.24)	2.62 (0.45–5.49)	0.6076
<3.40		30/42 (71.4 %)	52/82 (63.4 %)	0.4265	30/46 (65.2 %)	22/36 (61.1 %)	0.8180
IL-1β(pg/mL)	0.00–12.40	1.51 (0.75–2.77)	1.50 (0.69–2.40)	0.7354	1.37 (0.66–2.03)	1.81 (0.91–3.31)	0.1681
<12.40		42/42 (100 %)	82/82 (100 %)		46/46 (100 %)	36/36 (100 %)	
TNF-α(pg/mL)	0.00–4.60	1.27 (0.10–2.31)	1.23 (0.35–2.73)	0.6934	1.15 (0.59–2.77)	1.56 (0.14–2.74)	0.9032
<4.60		37/42 (88.1 %)	74/82 (90.2 %)	0.7609	42/46 (91.3 %)	32/36 (88.9 %)	0.7254
IFN-α(pg/mL)	0.00–8.50	2.24 (1.02–3.40)	2.23 (1.20–3.30)	0.2426	2.01 (1.11–2.82)	2.61 (1.37–3.36)	0.0670
<8.5		41/42 (97.6 %)	80/82 (97.6 %)	1.0000	44/46 (95.7 %)	36/36 (100 %)	0.5014
IFN-γ(pg/mL)	0.00–7.42	3.71 (0.10–15.67)	10.73 (3.04–27.38)	**0.0027**	13.16 (2.98–27.38)	10.05 (3.50–26.71)	0.6741
<7.42		26/42 (61.9 %)	30/82 (36.6 %)	**0.0083**	16/46 (34.8 %)	14/36 (38.9 %)	0.8180

Values are median (IQR) or n (%).

**Table 3:** The number of lymphocyte subsets evaluated among COVID-19 patients.

Variables	Range of normal value	Disease severity		*P*	In-hospital outcomes in severe		*P*
		Non-severe (n = 180)	Severe (n = 256)		Survivors (n = 170)	Non-survivors (n = 86)	
CD45^+^ cell (per μL)	1530–3700	972 (604–1350)	640 (389–1092)	**< 0.0001**	720 (437–1173)	502 (248–582)	**< 0.0001**
<1530		147 (81.7 %)	232 (90.6 %)	**0.009**	150 (88.2 %)	82 (95.3 %)	0.0722
CD3^+^ cell (per μL)	690–2540	374 (83–590)	217 (10–387)	**< 0.0001**	272 (55–458)	148 (10–276)	**< 0.0001**
<690		107 (59.4 %)	197 (77.0 %)	**0.0001**	122 (67.8 %）	75 (87.2 %)	0.0072
CD4^+^ cell (per μL)	410–1590	323 (214–587)	230 (125–407)	**< 0.0001**	273 (137–462)	168 (65–292)	**0.0002**
<410		114 (63.3 %)	193 (75.4 %)	**0.0077**	120 (70.6 %)	73 (84.9 %)	**0.0138**
CD8^+^ cell (per μL)	190–1140	235 (143–347)	140 (73–239)	**< 0.0001**	169 (87–284)	95 (53–152)	**< 0.0001**
<190		69 (38.3 %)	168 (65.6 %)	**< 0.0001**	94 (55.3 %)	74 (86.0 %)	**< 0.0001**
CD19^+^ cell (per μL)	90–660	118 (56–227)	88 (44–159)	**0.0009**	90 (44–169)	87 (44–135)	0.7342
<90		67 (37.2 %)	130 (50.8 %)	**0.0062**	136 (80.0 %)	70 (81.4 %)	0.8682
NK cell (per μL)	90–590	150 (97–262)	103 (56–193)	**< 0.0001**	133 (66–214)	74 (34–156)	**< 0.0001**
<90		38 (21.1 %)	117 (45.7 %)	**< 0.0001**	63 (37.1 %)	54 (62.3 %)	**0.0001**

Values are median (IQR) or n (%).

**Table 4:** The levels of immunoglobulin and complement factors among COVID-19 patients.

Variables	Range of normal value	Disease severity		*P*	In-hospital outcomes in severe		*P*
		Non-severe	Severe		Survivors	Non-survivors	
IgG (g/L)	7.00–16.00	11.88 ± 0.505	11.64 ± 0.414	0.7181	11.73 ± 0.569	11.48 ± 0.564	0.7678
<7		11/87 (12.6 %)	19/133 (14.3 %)	0.8416	11/84 (13.1 %)	7/49 (14.3 %)	1.0000
IgA(g/L)	0.70–4.00	2.432 ± 0.138	2.527 ± 0.109	0.5891	2.490 ± 0.132	2.591 ± 0.191	0.6566
<0.7		2/87 (2.3 %)	4/132 (3.0 %)	1.0000	3/84 (3.6 %)	1/48 (2.1 %)	1.0000
IgM(g/L)	0.40–2.30	1.003 ± 0.075	0.9055 ± 0.048	0.2552	0.9022 ± 0.058	0.9113 ± 0.086	0.9279
<0.4		7/87 (8.0 %)	13/132 (9.8 %)	0.8115	7/84 (8.3 %)	6/48 (12.5 %)	0.5459
C3 (g/L)	0.90–1.800	1.061 ± 0.034	0.9810 ± 0.026	0.0606	1.042 ± 0.032	0.8779 ± 0.041	**0.0021**
<0.9		23/86 (26.7 %)	38/126 (30.2 %)	0.6446	19/79 (24.1 %)	19/47 (40.4 %)	0.0707
C4 (g/L)	0.100–0.400	0.2858 ± 0.013	0.2727 ± 0.011	0.4467	0.2796 ± 0.013	0.2612 ± 0.020	0.4211
<0.1		3/85 (3.5 %)	10/126 (7.9 %)	0.2497	4/79 (5.1 %)	6/47 (12.8 %)	0.1729

Values are Mean ± SEM or n (%).

### 3.3 Potential markers of diagnostic value to distinguish non-severe and severe

**Fig. 1:**
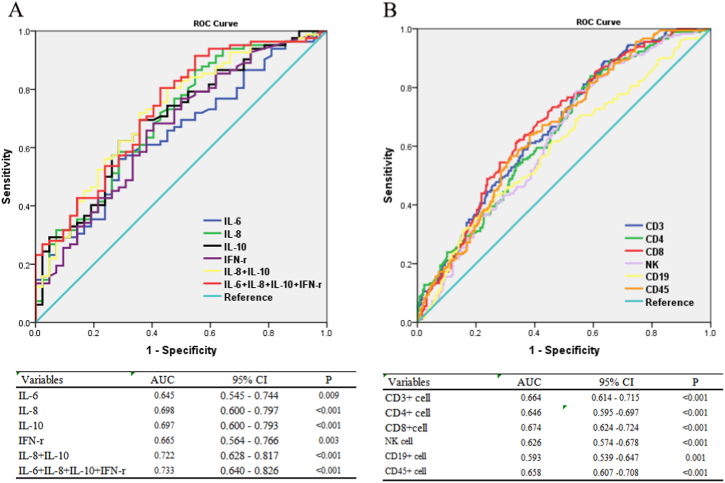
Potential markers of diagnostic value to distinguish non-severe and severe. ROC curve of inflammatory cytokines(A), and lymphocyte subset(B) to distinguish non-severe and severe.

To evaluate the diagnostic value of immunological factors in patients with non-severe and severe patients COVID-19, ROC curves and AUCs were shown in Fig. 1. As shown in Fig. 1A, for diagnosis of the severity of COVID-19, the area under ROC curve of IL-8 was the largest among all cytokines with the biggest AUC (0.698). And the AUCs of IL-6, IL-10 and IFN-γwere 0.645, 0.697 and 0.665, separately. The combined diagnosis model was established by logistic regression analysis, the area under ROC curve of combined all 4 cytokines increased to 0.733. In the lymphocyte subset group (Fig. 1B), the AUCs of CD3^+^ cell，CD4+ cell，CD8+ cell, NK cell, CD19^+^ cell and CD45^+^ cell were 0.664, 0.646, 0.674, 0.626, 0.593 and 0.658, respectively. Unfortunately, the AUC of combined index could not exceed the AUC of CD8^+^ cell (0.674), which did not shown in Figures.

### 3.4 Potential markers of diagnostic value to distinguish non-survivors between survivors

**Fig. 2:**
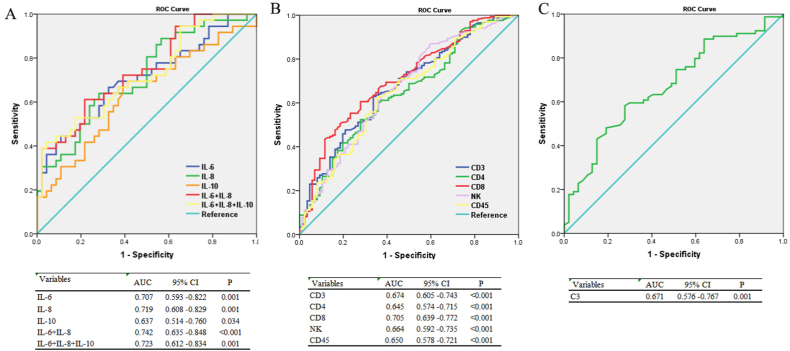
Potential markers of diagnostic value to distinguish non-survivors between survivors. ROC curve of inflammatory cytokines(A), lymphocyte subset(B), and immunoglobulin and complement factors (C) to distinguish non-survivors between survivors.

Further, the discrimination of the ability to make a distinction between survivors and non-survivors was evaluated with the area under the ROC curve. In the inflammatory cytokines group (Fig. 2A), the AUC of IL-8 was bigger than that of IL-6 and IL-10 (0.719 vs 0.707 and 0.637). Interestingly, the AUC of the combination of IL-8 and IL-6 was higher that of the combination of all three cytokines (0.742 vs 0.723). As shown in Fig. 2B, the AUC of CD8^+^ cell was the largest among all five lymphocyte subsets with the biggest AUC (0.705), which was higher than the AUC of CD3^+^ cell (0.674), CD4^+^ cell (0.645), NK (0.664) and CD45^+^ cell (0.650). There is no doubt that the AUC of combined index could not exceed the AUC of CD8^+^ cell, which did not shown in Figure. In the immunoglobulin and complement factors group (Fig. 2C), the AUC of C3 was 0.671.

### 3.5 Multivariate analysis of factors influencing the severity of COVID-19

**Fig. 3:**
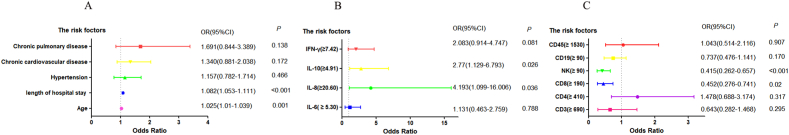
Multivariate analysis of factors influencing the severity of COVID-19. Binary Logistic regression analysis was performed in clinical characteristics of COVID-19 patients(A), inflammatory cytokines(B), and lymphocyte subset(C).

Binary Logistic regression analysis was performed with the severity of COVID-19 (non-severe = 0, severe = 1) as the dependent variable, and the statistically significant risk factors in univariate analysis as independent variables. As shown in Fig. 3A, length of hospital stay and age were independent risk factors for the severity of COVID-19. The result in Fig. 3B showed that patients with high levels of IL-8 and IL-10 were 4.193 times and 2.177 times respectively more likely to progress to a serious case than those with low levels of IL-8 and IL-10. The result in Fig. 3C showed that a higher frequency of CD8+cells and NK cell response was independently associated with less severe disease.

### 3.6 Multivariate analysis of factors influencing the in-hospital outcomes in severe COVID-19 patients

**Fig. 4:**
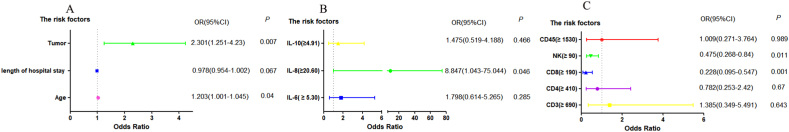
Multivariate analysis of factors influencing the in-hospital outcomes in severe COVID-19 patients. Binary Logistic regression analysis was performed in clinical characteristics of COVID-19 patients(A), inflammatory cytokines(B), and lymphocyte subset(C).

For the determination of in-hospital outcomes in severe COVID-19 patients, covariates were compared in survivors vs non-survivors. The result of Binary Logistic regression analysis shown that age and tumor were independent risk factors for death of COVID-19 patients (Fig. 4A). These patients with a high level of IL-8 was 8.847 times more likely to progress to death in severe case than those with low levels of IL-8 (Fig. 4B). The independent risk factors in lymphocyte subset group were a lower frequency of CD8^+^ cell and NK cell (Fig. 4C). Whereas a p value of lesser than 0.05 on the Hosmer-Lemeshow test in C3 group indicated poorness of fit.

### 3.7 Prognostic value of immunological factors on the day of admission

**Fig. 5:**
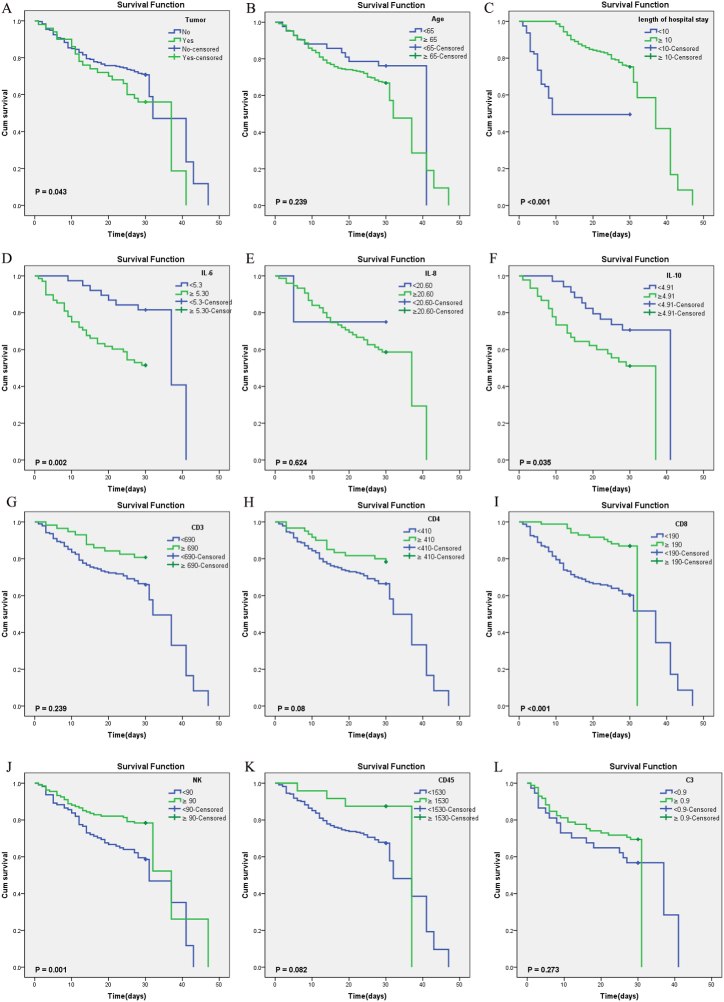
Prognostic value of immunological factors on the day of admission. Kaplan–Meier method and log-rank test were performed in factors (A-L).

Of the 290 severe patients admitted before February 7th, 101 (34.8 %) died within 30 days. Patients with tumor, or high IL-6 or elevated IL-10 expression exhibited a significantly shorter survival time (Fig. 5A, D, 5F). The study revealed a more positive prognosis for patients who experienced a prolonged hospital stay and an elevated levels of CD8^+^ cell and NK cell, as evidenced by the data presented in Fig. 5C, I, and 5J. There was no statistically significant disparity in prognosis observed between the older group (≥65 years old) and the younger group (<65 years old) as depicted in Fig. 5B. Meanwhile, no significant differences in prognosis were observed between patients with high and low IL-8 (Fig. 5E), CD3^+^ cell (Fig. 5G), CD4^+^ cell (Fig. 5H), CD45^+^ cell (Fig. 5K) or C3 levels (Fig.5L).

## 4 Discussion

On December 7, 2022, the Chinese government optimized the current epidemic prevention and control policy, and no longer adopted the Zero-COVID policy and mandatory. We retrospectively collected information on the first prevalence in Fujian after this policy, hoping to contribute to early identification of critical illness and guarantee the quality and safety of emergency in Fujian Province.

COVID-19 is associated with various forms of morbidity and complications. The infection can induce severe respiratory illnesses that are similar to severe acute respiratory syndrome coronavirus [2]. This study is intended to identify several immunological characteristics associated with disease progression and analyze the immune response in COVID-19. Our data demonstrates that in comparison with non-severe patients, severe patients are significantly older, with increased IL-6, IL-8, IL-10 and IFN-γ, and a lower lymphocyte subset. Non-survivors are older, having a higher level of IL-6 IL-8 and IL-10, decreased lymphocyte subset and C3 than survivors. In multivariate analysis, age, length of hospital stays, IL-8, IL-10, CD8 cell, and NK cell remain as significant predictors of severity, and age, tumor, IL-8, CD8 cell, and NK cell as significant predictors of mortality. Moreover, the exist of tumor, increased level of IL-6, IL-10, and decreased lymphocyte subset counts correlated to a poor outcome. Importantly, our observations indicate that immunological patterns are predictive and biomarkers of COVID-19 severity and mortality.

In our study, age difference between the cases was statistically significant. Furthermore, the results of binary logistic regression analysis suggested that elderly patients are more likely to develop severe cases and die. The age difference has also been found in other articles [[12], [13], [14], [15], [16]]. Moreover, it is worth noting that aging itself is a prominent risk factor for severe disease and death from COVID-19 [17,18]. In fact, the elderly patients have a high rate of non-specific complaints, lack of specificity in clinical manifestations, various underlying diseases, cognitive impairment, multi-drug sharing, organ aging, malnutrition, immune deficiency. This reminds us that as a vulnerable group of people, the elderly patients are prone to worsening of the disease. The government should encourage the elderly patients to minimize their participation in large gatherings, and improve the visiting system in places where the elderly patients gather such as apartments for the elderly patients.

The antibody response in COVID-19 is characterized by an increased IgM and IgG. IgA plays a crucial role in mucosal immunity [19]. Our data is in agreement with a study by Jing Liu, etc. [20] that shows the level of serum antibodies are irrelevant to severity and mortality. Due to the possible cross-reactions of binding antibodies with seasonal coronaviruses, serological tests are not useful for the early diagnosis of SARS-CoV-2 infections [19]. Besides, the virus-specific antibody response differs in patient with various severity. Specifically, survival patients have a common spike protein antibody response while a nucleocapsid-specific antibody response is common in non-survival patients [21]. The activation of the complement system suggests an overall beneficial effect against viruses, while it might also have toxic effects [22]. In particular, complement activation formed the basis of the pathophysiology in many lung diseases, such as asthma and acute respiratory distress syndrome (ARDS) [23]. COVID-19 patients exhibit extensive deposition of complement components within the lung septal microvasculature, resulting in upgraded C3 in serum [24,25]. In line with results from other study [26], we find elevated C3 level in survival patients in comparison with non-survival patients. Also, Delanghe et al. [27] found that C3 polymorphism was a significant determinant for COVID-19 mortality. Thus, C3 is considered an independent predictor of mortality for COVID-19.

Lymphocytopenia is widely considered to be correlated with the severity of COVID-19 [28,29], as our results suggest. Lymphocyte subsets are important factors for preserving immune function, which can be damaged by infection [30]. T-cell exhaustion, apoptosis, and a direct virus cytopathic effect were proposed to explain COVID-19-related lymphocytopenia [31]. Our result is consistent with a Meta analysis [32] selecting 20 publications and revealing that all lymphocytes types were significantly decreased in severe patients than non-severe patients. In asymptomatic patients, NK cells and antibodies help to control the infection. While protective memory occurs only in severe patients. The adaptive immune is much stronger in severe patients, which thus causes tissue damages and uncontrolled inflammatory reaction [33]. Lymphocytopenia may occur as a result of lymphocyte infiltration and sequestration in the lungs [34,35]. In non-survival patients, all lymphocyte types except CD19 cells, declined compared to survival patients, which consists with the level of antibody. CD19 expresses on all B-lymphocyte lineage cells (except for plasma cells), which induce SARS-CoV-2 neutralizing antibody response [36,37]. Studies suggest that disparate T and B cell responses could be due to a disconnect between B cell and T cell responses [38,39]. Therefore, these results suggest that absolute counts of major lymphocyte subsets are significantly and substantially decreased in severe COVID-19 disease and linked to patient outcomes.

Inflammatory cytokines were tested on admission. A number of inflammatory disease are mediated by an unbalance between pro-inflammatory and anti-inflammatory cytokines [40]. Pro-inflammatory cytokines (include IL-1β, IL-6, IL-8, IL-17, IFN-γ and TNF-α) mainly secreted by helper T (Th) 1 cells, CD4 T cell, activated macrophage and dendritic cells (DC) and play roles in activating various immune cells [41]. Regulatory T cells (Tregs), Th2 cells, activated macrophages, and monocytes are the main source of anti-inflammatory cytokines, such as IL-4, IL-10 and IL-13 [29]. In turn, inflammatory cytokines react on above and other immune cells. For instance, IFN-γ, secreted by NK cells and Th1, inhibits the replication of the virus and increases the cytotoxic T lymphocyte (CTL) killing activity in the body [42]. Excessive IL-6 may promote the development and differentiation of T cell subsets such as Th17, which secrets IL-17 that prevent virus-infected cells from apoptosis [43]. Evidence shows that both IL-6 and IL-8 are negatively correlated with NK cell and CD8 T cell [44]. Interestingly, there is a hypothesis that IL-10 and bacterial derivatives are related to the increased inflammatory [45]. Several studies highlighted that IL-6 IL-8 and IL-10 were significantly associated with a worse outcome [46,47]. An intense production of cytokines in infectious processes causes immunopathological reactions and result in serious complications, multiple organs damages or even death [48,49]. Comparable results were also revealed in our study that enhanced level of IL-6, IL-8, and IL-10 can be used as predictors for fast diagnosis of patients with higher risk of disease deterioration. Of note, IL-6 are recognized as a potential biomarker for development of fatal SARS-CoV-2 pneumonia [50,51]. Controversially, in various studies of COVID-19, the levels of interferon IFN-γ were significantly reduced in severe patients [52,53], while IFN-γ increased in other studies [20,47]. In our study, however, we observe higher IFN-γ level in severe patients compared to non-severe patients. Nevertheless, the role of IFN-γ is less clear [50]. IL-8 and IL-10 can be used to predict the infection severity. Meanwhile, an elevated level of IL-6 and IL-10 is associated with unfavorable prognosis for COVID-19 patients. As described above, inflammatory cytokines contributes to disease severity and mortality in COVID-19 patients.

Despite the widespread outbreak of the infection, a remarkable asymmetry is observed in the number of cases and in the distribution of the severity of the COVID-19 symptoms in patients with respect to the countries/regions [54]. Our data shows that the magnitude of cytokine storm is associated with the disease severity and mortality. Of note, decreased lymphocyte subsets and increased cytokines (IL-6, IL-8, IL-10 and IFN-γ) may aid the early identification of patients at risk of severe and critical COVID-19 and provide more definitive and timely approaches for treatment. The found may provide a basis for the prevention, control and management of the second COVID-19 pandemic in Fujian Province. Meanwhile, our study has some important limitations: it took place in a specific geographic area (one hospital in Fujian, China). Moreover, patients presented at various times during the disease with a varied base level of immunological characteristics in peripheral circulation.

## Ethics statement

This study was reviewed and approved by the Ethical Committee of Fujian Provincial Hospital, with the approval number: KY2021-03-013. All participants/patients provided informed consent to participate in the study.

## Data availability statement

Data associated with our study been deposited into a publicly available repository. Data will be made available on request.

## CRediT authorship contribution statement

**Yanli Kang:** Writing – review & editing, Writing – original draft, Formal analysis, Data curation. **Shifa Lu:** Writing – original draft, Data curation. **Ruifang Zhong:** Software, Data curation. **Jianbin You:** Formal analysis, Data curation. **Jiahao Chen:** Data curation. **Ling Li:** Formal analysis, Data curation. **Rongbin Huang:** Investigation, Data curation. **Yanyan Xie:** Data curation. **Falin Chen:** Data curation. **Jinhua Chen:** Supervision, Investigation, Conceptualization. **Liangyuan Chen:** Writing – review & editing, Visualization, Validation, Funding acquisition, Conceptualization.

## Declaration of competing interest

The authors declare that they have no known competing financial interests or personal relationships that could have appeared to influence the work reported in this paper.
